# SEM/TEM Investigation of Aluminide Coating Co-Doped with Pt and Hf Deposited on Inconel 625

**DOI:** 10.3390/ma11060898

**Published:** 2018-05-27

**Authors:** Jerzy Morgiel, Maryana Zagula-Yavorska, Maciej Zubko, Jolanta Romanowska

**Affiliations:** 1 Institute of Metallurgy and Materials Sciences, PAS, Reymonta 25 st., 30-059 Kraków, Poland; j.morgiel@imim.pl; 2 Faculty of Mechanical Engineering and Aeronautics, Rzeszow University of Technology, al. Powstańców Warszawy 12, 35-959 Rzeszów, Poland; jroman@prz.edu.pl; 3 Institute of Materials Science, University of Silesia, Pułku Piechoty 1a st., 41-500 Chorzów, Poland; maciej.zubko@us.edu.pl

**Keywords:** aluminide coating, hafnium, platinum, Inconel 625, transmission electron microscopy (TEM)

## Abstract

The effect of simultaneous introduction of Hf and Pt into aluminide coating deposited on Inconel 625 alloy was investigated using scanning and transmission electron microscopy (SEM/TEM) methods. The coating consisted of two layers: the additive and the interdiffusion. The additive layer and part of the interdiffusion layer consist of the β-NiAl type phase. The middle part of the interdiffusion layer comprised an interpenetrating finger-like structure formed by the β-NiAl and TCP—σ type phases with numerous fine Cr precipitates in the former and occasional larger precipitates of NbC carbides interspersed in between them. The σ type phase inclusions are situated at the border between the substrate and the interdiffusion layer. The experiment showed that platinum fully dissolves in the β-NiAl-type matrix, while most of the introduced hafnium accumulates in HfO_2_ dioxide precipitates located close to the additive/interdifusion interface.

## 1. Introduction

The hot section components of aircraft engines are exposed to an aggressive environment (high temperature oxidation and hot corrosion). Nickel-based superalloys have been widely used as structural materials in the hot section of aircraft engines [1]. The modification of chemical composition of nickel-based superalloys and coatings is used to protect superalloys against hot corrosion and high temperature oxidation. Advanced cooling schemes coupled with thermal barrier coatings (TBC) can enable the current families of superalloy components to meet the material needs for the engines of tomorrow [2]. Commercially advanced TBC systems are typically two-layered structures, consisting of a ceramic top coating and an underlying metallic bond coating [2]. The top coating, which is usually applied either by air plasma spraying (APS) or electron-beam physical vapor deposition (EB-PVD), is most often yttria-stabilized zirconia (YSZ). The YSZ top coating is also made “strain tolerant” by depositing a structure, that contains numerous pores and pathways. The consequent high oxygen permeability of the YSZ top coating imposes the constraint that the metallic bond coating must be resistant to oxidation attack. As oxygen may diffuse through the YSZ, even a fully dense structure (devoid of structural defects) would facilitate oxygen permeability to the bond coating. There are several types of bond coating—MCrAlY group coating (where M usually is Ni and/or Co.) and aluminide coatings based on the β-NiAl phase. Both the bond coatings are typically designed to provide oxidation resistance. However their needs are different. For instance in the case of the atmospheric plasma spraying (APS) TBCs, the top coating immediately delaminates during thermal cycling in the absence of the bond coating. In this case the bond coating also serves to ensure top coating adhesion, mitigates thermal mismatch stresses with the substrate and is not solely for oxidation protection. The compatibility between the thermally grown oxide (TGO) scale (comprising of Al_2_O_3_ and other oxides) forms during oxidation and TBCs plays a crucial role in governing coatings durability [3]. Leckie et al. [4] studied the thermochemical compatibility between alumina (TGO) and Gd_2_Zr_2_O_7_. They reported a growing GdAl_2_O_3_ interphase at the Gd_2_Zr_2_O_7_—TGO interface compromising the integrity of the TBCs. MCrAlY group bond coatings are typically applied by using thermal spraying processes, such as high-velocity oxygen fuel spraying (HVOF) [5], low-pressure plasma spraying (LPPS) [6] or APS [7]. During oxidation of this type of bond coatings the oxide layer has a heterogeneous morphology. The reason for the inhomogeneity of the oxide layer is that MCrAlY coating is composed of two phases—β-NiAl and γ-Ni. Upon oxidation all phases present in the bond coating will tend to form their own oxides and next will form homogeneous Al_2_O_3_. Oxide phase transformation induces stresses and leads to cracks formation in the oxide layer. 

Oxidation of the aluminide coating containing the β-NiAl phase leads to formation of slow growing and homogeneous Al_2_O_3_. This oxide layer formed on the surface of the aluminide coating is more adherent than the layer formed on MCrAlY coating because of the lack of the heterogeneous oxide phase transformation. Aluminide coatings are produced by different methods: pack cementation, “out of pack” and chemical vapor deposition [3]. 

Addition of platinum to aluminide coatings leads to improvement of their performance [8], namely oxidation resistance via number of mechanisms [1,9]. Platinum lowers the activity of aluminium in the NiAl phase and promotes alumina scale formation during the oxidation of coated superalloys. Moreover, it improves scale adhesion by inhibiting sulphur segregation to the scale/coating interface [10]. That is why the thermal cycling lifetime of platinum modified aluminide coated superalloys is longer than simple aluminide-coated superalloys. 

Platinum could be introduced into aluminide coatings on the superalloys by electroplating of their surface followed by aluminizing by pack cementation, above-the-pack or chemical vapor deposition (CVD) processes [11]. The microstructure of Pt modified coatings consists of the ζ-PtAl_2_ and β-NiAl phases [11]. 

Research performed on Hf-doped cast alloys indicate that its additions to β-NiAl or Ni-Pt phases reduces their oxidation parabolic rate constant by a factor of 10 [12]. Izumi et al. [13] showed that γ + γ′ Ni-20Al-20Pt + Hf and Ni-20Al-22Pt-7Co-7Cr-0.7Hf cast alloys form adherent α-Al_2_O_3_ scales with significantly reduced spallation caused by cyclic oxidation. It also agrees with the earlier findings that introduction of small hafnium additions to nickel [14] and cobalt-based superalloys [15] improves adherence and durability of the protective alumina scale on their coatings. 

Simultaneous ‘co-doping’ using Pt and Hf is much less understood, but even a few papers published up till now indicate that it may be a promising way of protecting nickel-based alloys from high temperature oxidation. Nesbitt et al. [16] and Warnes et al. [17] reported that NiAl-based alloys containing a small amount of hafnium (<1 wt %) added with or in place of platinum could significantly extend the superalloys’ service lifetime. Similar Pt-(CoNi)Al and Hf-Pt-(CoNi)Al coatings on superalloys [18] have better oxidation resistance than (CoNi)Al and Hf-(CoNi)Al ones. Of all the investigated cases, high-temperature corrosion of Hf-Pt-(CoNi)Al resulted in growth of denser scale and lower spallation. The synergistic effect of Hf and Pt on the oxidation resistance of single crystal superalloys was described by Yang et al. [19]. The alumina scale formed on the Hf-doped coating during oxidation was much thinner than on normal (Ni,Pt)Al coatings. That is evidence that Hf reduced the oxide growth rate. Hafnium tends to occupy the preferred site of aluminum and follows a similar diffusion path to alumina. Thus, the diffusion of aluminum in alumina scale should be affected by hafnium. Hafnium possesses higher values of diffusion activation energy and a lower diffusivity constant than aluminum. Therefore, hafnium diffuses much slower than aluminum. Thus, hafnium atoms could act as partial path blocker for aluminum diffusion within the Al_2_O_3_ scale and could decrease Al_2_O_3_ scale growth. The alumina scale formed on Hf-doped (Ni,Pt)Al coating is much smoother than on the (Ni,Pt)Al one. This is advantageous for acquiring a prolonged service life for coatings. 

Hafnium may be introduced to the coating either by diffusion from substrates or as a part of its deposition process including CVD and pack cementation [12,14,15]. The latter two ways are more efficient and, therefore, more practical in industrial applications. Kim et al. [14] determined HfCl_3_/AlCl_3_ activator ratios allowing various hafnium ratios to be obtained in as deposited material. Experiments performed at Steel Improvement and Forge Company (SIFCO) [12] fully confirmed that hafnium could indeed be incorporated into aluminide coatings deposited on Ni-base superalloys with an industrial vapor phase deposition systems. However, there is no experimental documentation of the structure of the aluminide coating co-doped with both Pt and Hf.

Widely used nickel superalloys such as CMSX 4, PWA 1484, CMSX 10 and Rene N6 contain a small amount of sulfur. These superalloys contain 3% to 6 wt % of rhenium (refractory, high-melting element). The aluminum concentration is approximately 6 wt %. It is not enough to provide good oxidation resistance at the operating temperature. Therefore, the platinum modified aluminide bond coating are deposited. However CMSX 4, PWA 1484, CMSX 10 and Rene N6 contain small amount of hafnium. Moreover the microstructure of aluminide coatings deposited on CMSX 4, PWA 1484, CMSX 10 and Rene N6 is known [12]. 

Inconel 625 was chosen for this study because it does not contain Hf that might obscure Hf effects from the coating. Moreover scanning and transmission electron microscopy (SEM/TEM) investigation of coatings deposited on Inconel 625 has not been performed yet. 

Therefore, the aim of this paper was to investigate a Pt and Hf co-doped coating deposited on Inconel 625 alloy using SEM/TEM methods. This kind of coating was analysed by Yang et al. [19]. They found hafnium-rich inclusions in the belt between additive and interdiffusion zones. They suggested that this hafnium-rich belt may be a hafnium reservoir. Moreover, hafnium could be dissolved in the coating. This suggestion was proposed without the analysis of chemical and phase composition of hafnium-rich precipitates. That is why the authors of this paper decided to analyze the structure of the coating and chemical and phase composition of these hafnium-rich precipitates. 

## 2. Experimental Methods

Samples made of Inconel 625 alloy (chemical composition presented in Table 1) were ground using SiC No 600, degreased in ethanol, ultrasonically cleaned and finally electroplated with 5 μm thick platinum layer. Samples were ground using SiC 600 to make the platinum deposition process more efficient. Sample surface preparation to the platinum electroplating process was performed according to the procedure accepted by the National Aerospace and Defense Contractors Accreditation Program. Platinum electroplating was preceded by activation. The activation was performed for 1 h by means of tetraammineplatinum (II) composite bath—Pt(NH_3_)_2_(NO_2_)_2_ 15 g/dm^3^, which enables the rapid growth of an adherent and relatively homogeneous coating. The electroplating was performed with the current density of 0.1 A/dm^2^ [11].

The aluminide coatings were deposited by the CVD method using a BPXPR0325S equipment manufactured by the IonBond company. The applied CVD aluminizing and hafnizing protocol was established through a trial and error methodology. The details of applied CVD aluminizing and hafnizing processes were as follows:

- heating from the room temperature up to 1040 °C;
- aluminizing at 1040 °C for 60 min;
- aluminizing with hafnizing at 1040 °C for 540 min;
- aluminizing at 1040 °C for 60 min;
- cooling samples with the furnace.

The thin foils for TEM/Bright Field (BF) observations (25 mm × 15 mm × 100 nm) were cut out using focus Ga+ ion beam. 

**Figure 1:**
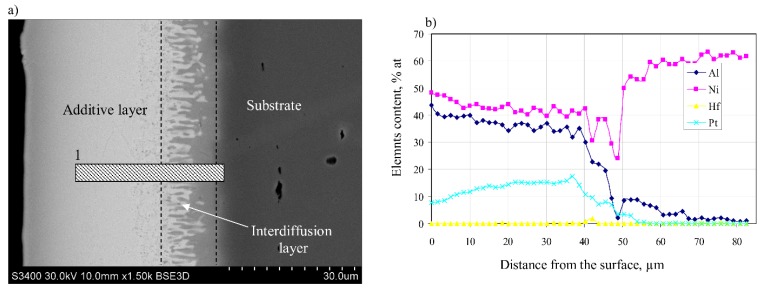
The microstructure of hafnium and platinum-modified aluminide coating deposited by the chemical vapor deposition (CVD) method on the Inconel 625 alloy (striped bar presents an area from which a thin foil for transmission electron microscope (TEM) observations was cut-out) (**a**); and the elements’ content on the cross-section of coating (**b**).

The microstructure and local chemical analysis was performed using Tecnai G2 SuperTWINN FEG (200 kV) TEM (Fei, Hillsboro, OR, USA) equipped with the X-ray energy dispersive spectroscope (EDS) and high-angle annular dark field (HAADF) detectors. The phase analysis including precession experiments were undertaken with a JEM 3010 TEM equipped with DigiSTAR developed by NanoMEGAS company (Forest, Belgium). Cell parameters of investigated phases were determined ab initio by the 3D diffraction tomography through the following procedure: (a) series of precession diffraction patterns were collected with precession angle of 1° covering angular range of 56°; (b) based on recorded pattern reconstruction of 3D reciprocal space and (c) 3D diffraction peaks positions were determined; (d) in order to cover the diffraction, missing cone differential vector space was created and (e) cluster analysis was performed; (f) based on the cluster centers, the unit cell was determined. The above analysis of the 3D diffraction tomography measurements were performed using ADT3D software (Jeol, Tokyo, Japan). Thin foils for all TEM investigations were obtained from the section of the coating/substrate interface (i.e., perpendicularly to the surface as presented in Figure 1) using the Quanta dual beam focused ion beam (FIB) (Fei, Hillsboro, OR, USA) equipped with an Omniprobe lift-out system.

## 3. Results and Discussion

The cross-section SEM image (Figure 1a) indicates that hafnium and platinum-modified aluminide coating deposited on Inconel 625 alloy consists of two layers: the additive (35 μm thick) and the interdiffusion one (14 μm thick), respectively. There is a generally accepted division of aluminide coatings into additive and interdiffusion layers [12,17,18,19]. The thickness of hafnium and platinum-modified aluminide coating is 49 μm. The area marked 1 in Figure 1a was chosen for cutting thin foil using the FIB technique. EDS analysis on the cross-section showed that the top, additive layer is composed of the β-(Ni,Pt)Al phase (Figure 1b).

**Figure 2:**
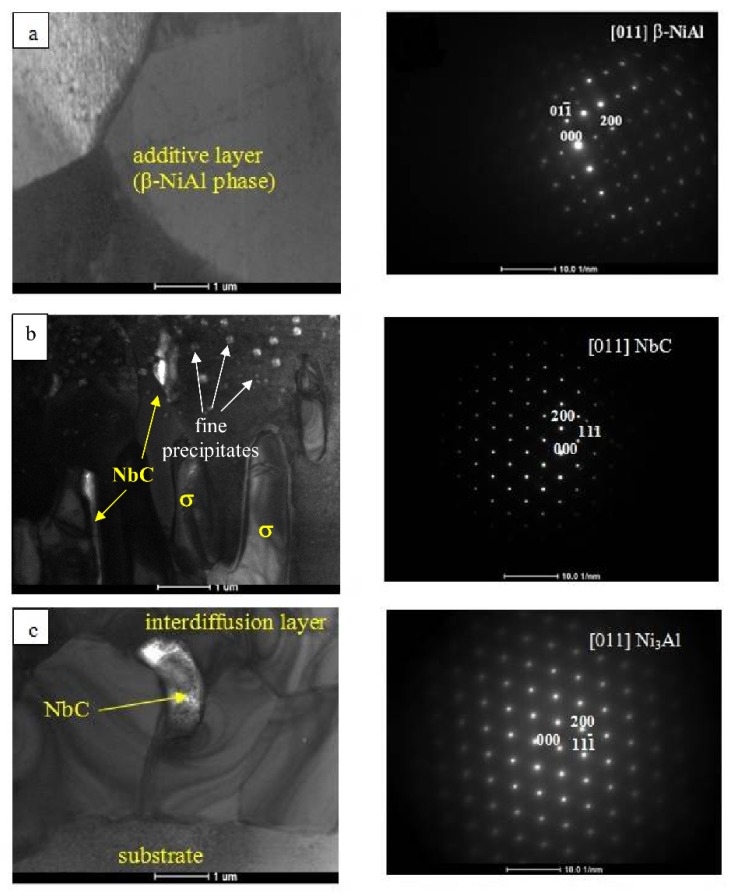
TEM images of the cross-section of the Hf and Pt modified aluminide coating: (**a**) the additive layer; (**b**) the interdiffusion layer; (**c**) the interdiffusion layer/substrate interface and accompanying electron diffraction patterns.

The electron diffraction patterns proved the presence of the β-NiAl type phase both in the additive layer and in the upper part of the interdiffusion zone (Figure 2a,b).

The following TEM observations of the cross-section of this coating helped to establish that the additive layer was built of coarse equiaxed grains (Figure 2a). The interdiffusion zone also starts with the same type of grains but with numerous fine precipitates (Figure 2b). Precipitates located deeper have greater density, while the matrix forms an interpenetrating finger-like microstructure with a new phase characterized by its practically defect-free lattice (aside from occasional stacking faults). 

**Figure 3:**
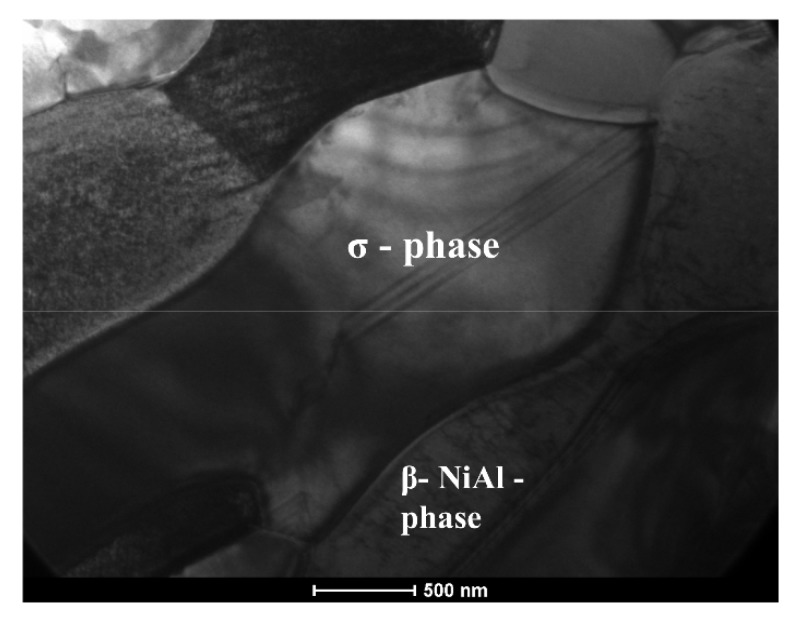
TEM images of the cross-section of the Hf and Pt-modified aluminide coating, σ and β-NiAl phase.

This new phase forms a continuous layer at the bottom of the interdiffusion layer, i.e., at the interface with the substrate. Within the area where NiAl and the new phase interpenetrates, a presence of NbC carbides were occasionally noted (Figure 2b,c). The sigma phase visible in Figure 2b as finger-like features shows contrast changes connected with the presence of extinction lines, which are very sensitive to any defects. However, presently they only occasionally carry sets of parallel lines elucidating the presence of stacking faults (crystallite in the centre). On the other hand, the neighboring crystallite of β-NiAl phase located just below shows the presence of numerous dislocations (visible as corrugated short lines) (Figure 3). Therefore, the microstructure features allow them to be easily distinguished without solving electron diffraction.

The selected area electron diffraction patterns proved the presence of the β-NiAl type phase both in the additive layer and an upper part of the interdiffusion zone. However, determining a crystal structure of finger-like precipitates extending from the bottom—due to their possible multi-component chemical composition—posed a serious problem. As the solubility of refractory elements in the β-NiAl interdiffusion layer is generally very low, so they usually form chromium, molybdenum and niobium carbides [20,21,22]. An investigation of the effect of the exposure of Inconel 617 superalloy to high temperature (>800 °C) showed that up to three types of carbides can co-exist, i.e., Cr-rich M_23_C_6_, Mo-rich M_6_C and Nb-rich MC [20,21]. Simultaneously, a preferential precipitation of Cr-rich M_23_C_6_ type carbide (Cr_23_C_6_) appears to be more probable than Mo-rich M_6_C carbide (Mo_6_C) due to a higher diffusion coefficient of Cr than Mo in Ni-base superalloys [22]. The crystal structure and chemical composition of phases present in Inconel 625 superalloy, i.e., MC, M_6_C, M_23_C_6_ carbides, γ″, δ, Laves and (Cr,Nb)_2_N formed at temperatures ranging from 540 to 1100 °C, were given by Floreen et al. [22]. 

The electron diffraction patterns acquired from the finger-like precipitates from the interdiffusion layer excluded, however, the presence of any of the phases, listed by Floreen et al. [22]. 

**Figure 4:**
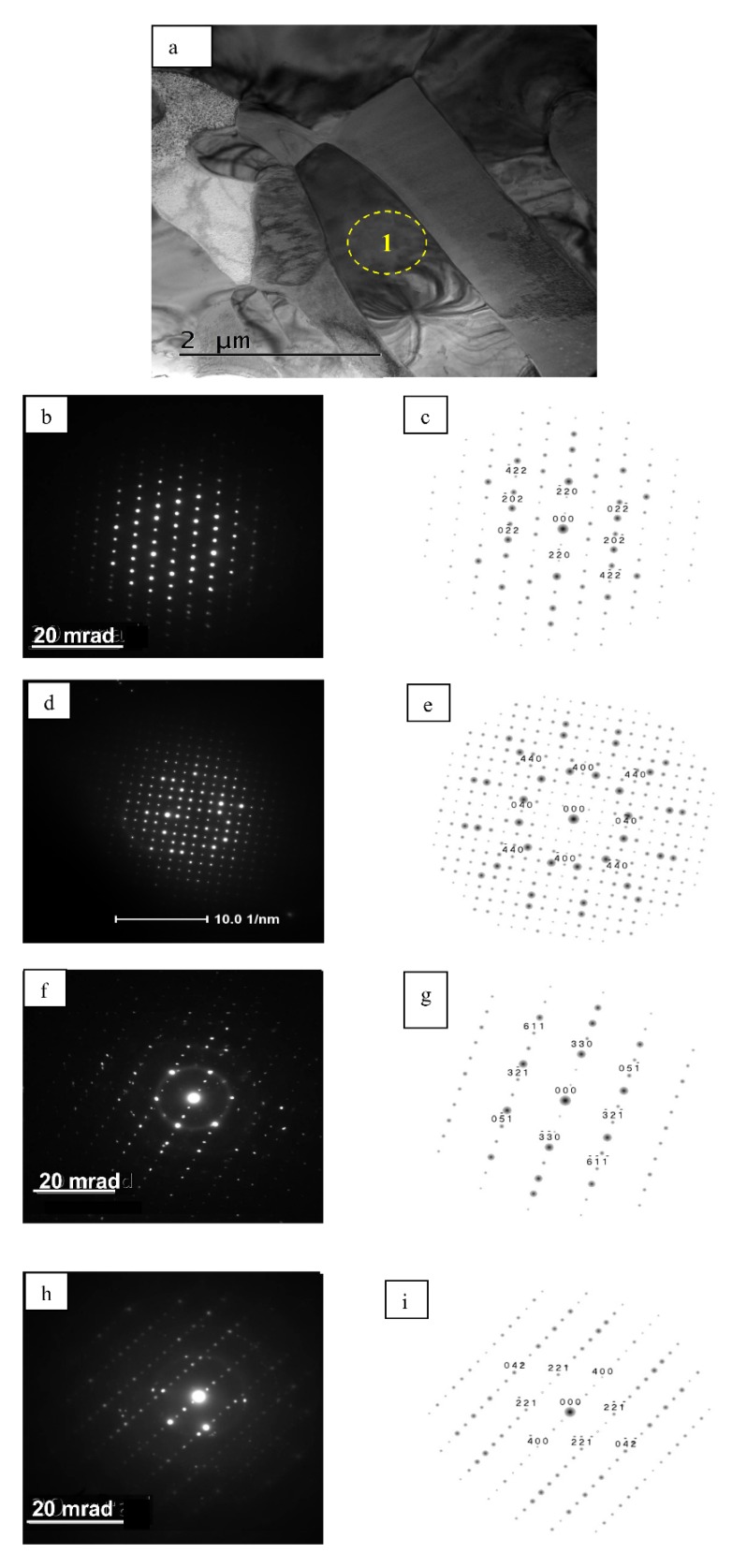
TEM image of cross-section of precipitates in the interdiffusion layer (**a**); and a series of electron diffractions taken at different tilt angles (**b**,**d**,**f**,**h**) with their solutions (**c**,**e**,**g**,**i**), respectively.

Difractions presented in Figure 4 were obtained by the precession electron diffraction technique. Their analysis indicated that the investigated precipitates are of a tetragonal structure, with unit cell parameters equal to: a_0_ = 0.954 nm, c_0_ = 0.491 nm (being significantly bigger than a typical σ—Cr_7_Ni_3_ phase: a_0_ = 0.871 nm, c_0_ = 0.449 nm). On the other hand, cell parameters of the observed phase are similar to that of the σ phase in nickel superalloys (a_0_ = 0.93 nm, c_0_ = 0.49 nm) [23]. The EDS estimated chemical composition of the investigated σ type phase was as follows: ~50 at % Cr, ~25 at % Ni, ~17 at % Mo, ~6 at % Fe, ~2 at % Pt, while the β-NiAl type phase contained ~50 at % Ni, 30 at % Al and 20 at % Pt (the presence of Cr peak in the latter spectra, of up to a few % strength, resulted from beam overlapping of chromium-rich precipitates and, therefore, was omitted in calculations) (Table 2). 

**Figure 5:**
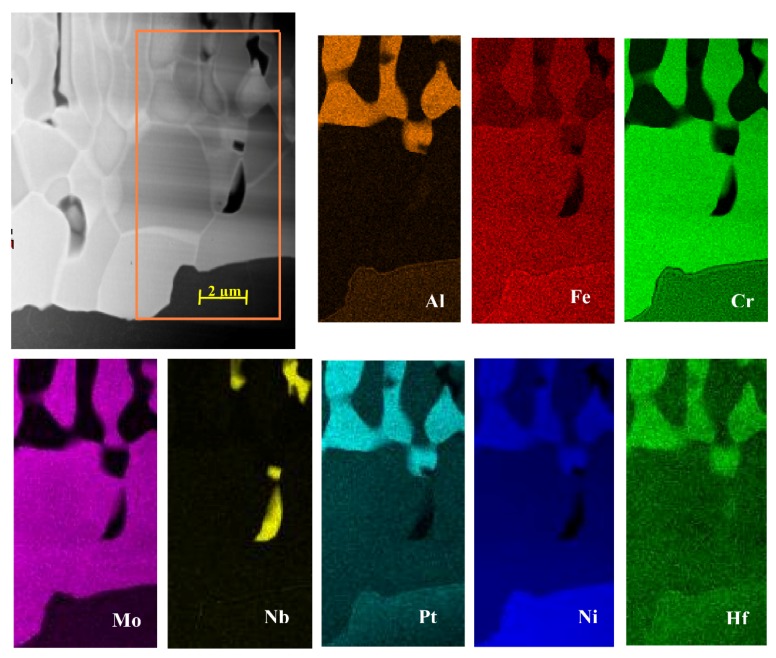
Scanning Transmission Electron Microscopy (STEM) high-angle annular dark field (HAADF) image of the interdiffusion layer with corresponding maps presenting distribution of Al, Cr, Fe, Hf, Mo, Nb, Ni, Pt.

**Figure 6:**
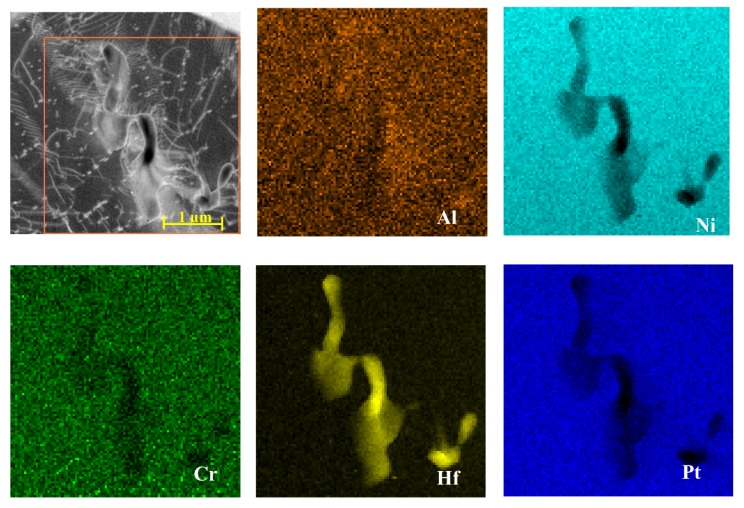
STEM HAADF image of the additive layer with corresponding maps presenting distribution of Al, Ni, Cr, Hf, Pt (Run I).

**Figure 7:**
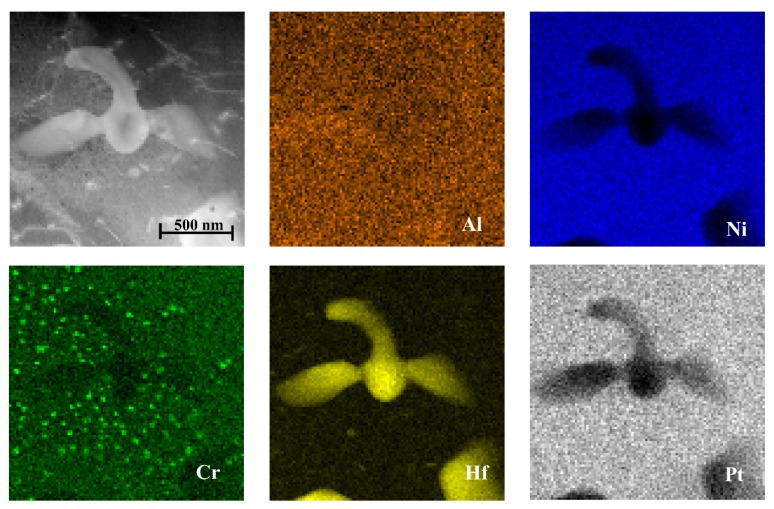
STEM HAADF image of the additive layer with corresponding maps presenting distribution of Al, Ni, Cr, Hf, Pt (Run II).

The maps of local chemical composition revealed a uniform distribution of nickel and aluminium in the additive and upper part of the interdiffusion layers, confirming their same phase composition (Figure 5, Figure 6 and Figure 7). 

**Figure 8:**
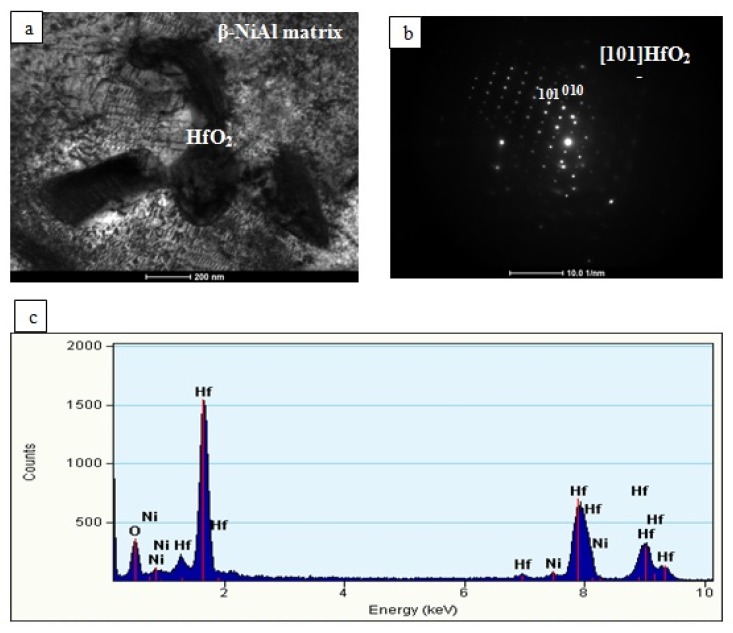
TEM image of the hafnium-rich particle (**a**) with accompanying diffraction (**b**) and (**c**) energy dispersive spectroscope (EDS) spectra.

Platinum dissolved in the β-NiAl phase in the additive and interdiffusion layers. Hafnium was present there only in the form of Hf-rich precipitates of size from ~0.5 to ~1 μm located mainly in at the bottom of the additive/top of the interdiffusion layer area (Figure 6 and Figure 7). The EDS spectra acquired from it as well as acquired electron diffraction patterns helped to prove it as HfO_2_ oxide (Figure 8). Its location in this area could be explained by scavenging by surplus hafnium of any oxide remnants present during the CVD deposition process. Such surplus is a direct consequence of fast saturation of the coating with hafnium and its low diffusivity in the β-NiAl type phase [24].

Outward nickel and inward aluminum diffusion leads to formation of the β-NiAl-type phase both in the additive and top interdiffusion layers. Moreover, niobium forms NbC precipitates in the interdiffusion layer. Neither Kirkendall-like porosity nor any Al_2_O_3_ oxide precipitates at the additive/interdiffusion layer interface were observed, even as they were noted at such interfaces in case of hafnium-doped aluminide coatings on pure nickel [25]. Kirkendall porosity forms in some systems because of unbalanced diffusion of species into and out of the alloy. The area of significant porosity is formed between the additive and interdiffusion layers of the aluminide coating. According to Kirkendall–Frenkel theory, there is an unbalanced flow of nickel and aluminum atoms in the diffusion zone. The value of the nickel diffusion coefficient is bigger than aluminum diffusion coefficient (*D*_Ni_ *> D*_Al_). The unbalanced flux of nickel and aluminum atoms is caused by the differences in microvolume and causes stress in the diffusion zone. The microvolume is reduced in the area of higher nickel concentration and vacancies are formed. When the number of vacancies is big, vacancies coalescence and this way pores are formed. This way Kirkendall porosity is generated [26]. 

As no Hf-rich intermetallic phases, for instance Hf_3_Ni, HfNi_3_, Hf_2_Ni_7_ or Ni_2_AlHf, were noted in the interdiffusion layer, this means that Hf diffusion in the aluminide layer is limited. 

The coating’s formation seems to be a result of several processes [27]:

- diffusion of nickel, chromium, molybdenum, iron and niobium from the substrate to the surface, leading to formation of the σ phase and NbC carbides in the interdiffusion layer;
- diffusion of aluminium from the gas phase to the alloy surface where platinum is diluted by nickel. Since platinum has more affinity to nickel, it forms the (Ni,Pt)Al phase where platinum is in substitutional solid solution in the additive layer.

The Hf-rich inclusions are distributed at the additive/interdiffusion layer interface. This is evidence that Hf deposition occurs after 60 min of aluminizing. The low hafnium diffusivity and solubility in the β-NiAl is reflected in the distribution of Hf-rich inclusions. They are situated at the additive/interdiffusion layer interface, which corresponds to the initial surface of the substrate. The diffusion activation energy of hafnium is higher than those of platinum, nickel, and aluminum, but the diffusivity is lower [19]. Therefore, the location of Hf incluions remains the same during the coating’s growth; only at the additive/interdiffusion layer interface. They do not diffuse to the interdiffusion layer nor to the surface of the coating. Similar results were also obtained by Wang et al. [12].

Yang et al. [19] compared oxidation resistance of Hf-doped single-phase Pt-modified aluminide coating and undoped single-phase Pt-modified aluminide coating and found that the hafnium-doped one has better oxidation resistance than the undoped one. 

An oxidation test was performed by Yang et al. [19]. Therefore, the authors of this manuscript did not perform an oxidation test again. Authors of this manuscript paid attention to the suggestion, proposed in Yang et al. [19], about Hf-rich inclusions and their role in the improvement of oxidation resistance. 

Yang et al. [19] revealed the presence of the Hf-rich belt at the additive/interdiffusion layer interface of the Hf-doped single-phase Pt-modified aluminide coating on the nickel-based single crystal (SC) superalloy. They suggest that the Hf-rich belt could act as the hafnium reservoir. During oxidation the internal Hf-rich precipitates could be dissolved at a certain rate driven by the chemical potential of the composition gradient. Because the solubility of Hf in β-NiAl phase is very low, less than 0.05 at % [19], the degradation rate of the Hf-rich belts was moderate as the outer (Ni,Pt)Al could not accommodate a higher content of Hf.

This suggestion was proposed without the analysis of chemical and phase composition of hafnium-rich precipitates. Our research revealed the presence of nanosize HfO_2_ particles. Therefore, we assume that these HfO_2_ particles cannot be the hafnium reservoir nor can they dissolve in the matrix. Nevertheless, they improve oxidation resistance [19], but the mechanism of this improvement must be different than suggested by Yang et al. [19]

The authors of this manuscript suggest, that HfO_2_ nanoparticles distributed at the interdiffusion/addition layer border retard the diffusion of oxygen and this way oxidation is slowed down. 

## 4. Conclusions

The SEM/TEM investigation of the microstructure and phase composition of hafnium and platinum co-doped aluminide coating deposited on Inconel 625 alloy proved that:

1. The coating consists of the additive and the interdiffusion layers, of which the first and the adjacent area of the second are built of the β-NiAl phase. The bottom part of the interdiffusion layer is formed by a layer of σ-type phase, while its middle part fills in an interpenetrating finger-like microstructure of σ-phase and β-NiAl with occasional NbC precipitates.
2. The TCP σ-type phase was found to be characterized by a tetragonal unit cell of a_0_ = 0.954 nm and c_0_ = 0.491 nm. Its chemical composition was estimated at 49.5 at % Cr, 24.8 at % Ni, 17.5 at % Mo, 6.5 at % Fe, 1.7 at % Pt.
3. The growth of the aluminide coating is controlled by outward diffusion of nickel from the substrate accompanied by simultaneous inward diffusion of aluminum. The growth of the σ-type precipitates into a continuous layer at the bottom of the interdiffusion layer effectively slows down both processes.
4. Platinum was found to dissolve in the β-NiAl phase present in both layers up to 20 at %, while the EDS measurable amount of hafnium was found only in HfO_2_ dioxide inclusions distributed close to the boundary between the additive and interdiffusion layers.

## Figures and Tables

**Table 1:** Chemical composition of Inconel 625 alloy, wt %.

Ni	Cr	C	Mo	Nb	Ta	Al	Ti	Co.	Fe	S	Si
59.5	21.5	0.1	9	2.0	0.5	0.4	0.485	1	5	0.015	0.5

**Table 2:** Chemical composition of σ and β-NiAl type phase, at %.

Phase	Ni	Cr	Mo	Fe	Pt	Al
σ	24.8	49.5	17.5	6.5	1.7	-
β-NiAl	50	-	-	-	20	30
